# Differential Impact of WM Load on Attention in Young Adults Versus Children and Adolescents

**DOI:** 10.3390/children11091057

**Published:** 2024-08-29

**Authors:** Hyojin Park, So-Yeon Kim

**Affiliations:** Department of Psychology, Faculty of Psychology, Duksung Women’s University, Seoul 01369, Republic of Korea; gywls6512@duksung.ac.kr

**Keywords:** selective attention, working memory, children, cognitive development, Stroop interference

## Abstract

**Background:** This study aimed to examine how concurrent working memory (WM) loads affect selective attention, and to explore developmental differences between young adults and children/adolescents aged 10 to 14 years. **Methods**: We employed a modified Stroop task with verbal or spatial WM loads to assess their impact on attention. **Results:** In adults, we found increased Stroop effects when WM load overlapped with target processing and decreased Stroop effects when WM load overlapped with distractor processing. Conversely, in children/adolescents, WM loads did not significantly impact target or distractor processing, indicating no change in Stroop effects under dual-task conditions. Interestingly, results from the correlational analyses revealed that as participants’ ages increase, the interference effect under the WM load that shares resources with distractor processing in the attention task decreases. **Conclusions:** Our findings suggest that the interaction between attention and WM differs across developmental stages. While adults showed distinctive effects of concurrent WM loads on attention processing depending on the cognitive resources utilized, children/adolescents failed to show the interaction between the two cognitive systems. Furthermore, a significant relationship between age and the effects of WM load on attention was observed, providing insights into the development of the interaction between WM and attention.

## 1. Introduction

Working memory (WM) plays a crucial role in the development of cognitive skills in school-aged children, including reading, mathematical ability, social skills, general intelligence, and overall academic achievement [1,2]. WM achieves this by temporarily storing and W manipulating information necessary for goal-directed behaviors [3]. Neuroimaging research on WM has demonstrated that the neural correlates of WM continue to develop from childhood through adolescence and into young adulthood [4,5].

Since both WM and selective attention require effortful processing and are essential for goal-directed behaviors such as academic performance, their relationship has been extensively studied in adults using a dual-task paradigm [6,7,8,9]. Previous research has shown that selective attention is hampered by high WM loads [8] or limited WM capacities [10]. Studies utilizing a dual-task paradigm have suggested that the burden of WM increases distractor processing in tasks such as the flanker task, causing impairment in attention processing [8,10,11]. However, the effects of WM load can vary depending on the types of WM load [7,9,12,13]. WM has distinct storage processes for phonological information and visuo-spatial information, so the WM load may not cause interference unless it overlaps with the processing required for the task [9]. Moreover, when the contents of the WM task overlap more with the processing of the disturbance stimulus than the target stimulus, the high WM load can reduce the processing of the distractor stimulus, thereby improving performance in the attention task in general [7,13].

Kim et al. [7] conducted one of the early studies on the distinctive interaction effects between WM and selective attention. Using a dual-task paradigm, they explored how the type of resource filling the WM capacity and the resource required for processing the target or distractor stimuli in the attention task led to variations in Stroop interference effects. In their first experiment, participants were tasked with remembering seven syllables in WM while performing a meaning comparison Stroop task. When the verbal resource used in WM overlapped with target processing in the Stroop task, the Stroop effect increased, resulting in slower reaction times for target detection. In their second experiment, participants were involved in the same verbal WM task, but the concurrent attention task was changed to a color comparison Stroop task, where the distractor processing required verbal resources. Using this manipulation to align resources for WM and distractor processing in the attention task, the researchers found that performance in the attention task improved under the dual-task condition. Overall, Kim and colleagues [7] provided novel evidence on the interaction effects between WM and attention, emphasizing the crucial role of the type of resources shared between the two cognitive processes.

Similarly, Zhao and colleagues [14] attempted to segment the resources of WM to examine how the load of a specific WM affected the Simon effect. The Simon effect refers to the phenomenon where participants react more slowly when a stimulus appears in a spatially incongruent location compared to a congruent location, where the stimulus and the required response are aligned (e.g., a red color associated with left response and a green color associated with right response) [15]. That is, the spatial WM load interfered with the Simon task requiring spatial resources, whereas the verbal WM load did not affect the same Simon task. In a later study [16], the researchers conducted a color-discrimination Stroop task involving verbal resources for distractor processing under a dual-task condition with a spatial WM load. The authors argued that Chinese characters were processed by their shape first, rather than their meaning, causing spatial WM to overlap with distractor processing. As a result, the distraction effect decreased under the dual-task condition compared to the single-task condition. Furthermore, using electroencephalography (EEG), the researchers demonstrated that theta power in the central–frontal region of the brain, associated with executive function, decreased in the dual-task condition. They also found a positive correlation between the reduction in theta activity and the decrease in the interreference effect. In line with findings from Kim et al. [7], these results add behavioral and neurophysiological evidence on the differentiated effects of WM load on attention processes, depending on the overlapping resources used in both processes.

It is noteworthy that the interaction between WM and attention has been investigated primarily in adult populations. In other words, there is relatively insufficient evidence on the impact of WM load on selective attention in typically developing children and adolescents compared to the research in young adults. To explore the development of the interaction between WM and selective attention, it is essential to first determine the developmental progression of each element.

From childhood though adolescence, there is a period of rapid development in WM, observable through behavioral indicators such as faster response times and increased accuracy in task performance. Additionally, neurobiological indicators, such as increased activation of brain regions involved in WM processing, further illustrate this development [17,18]. For WM maintenance, which requires storing information for a short period, accuracy reaches adult-like levels by 10 to 12 years of age. However, the ability to maintain and manipulate information in WM continues to develop into adolescence [4,5,19]. Notably, the ventrolateral prefrontal cortex (VLPFC), strongly associated with WM maintenance, develops at earlier ages than the dorsolateral prefrontal cortex (DLPFC), which is linked to WM manipulation [5,17]. This developmental trajectory can reveal differences in activation patterns between children and adults. In a study by Jolles and colleagues [4] comparing 11–13-year-old children and adults, both age and performance improvements in accuracy and response times were noted. The study found that activation in the left VLPFC, related to WM maintenance, did not differ between children and adults. However, in the right DLPFC, activation was stronger in adults compared to children, especially during performance requiring WM manipulation.

Understanding the development of selective attention is also noteworthy before investigating the interaction between attention and WM in childhood. It has been shown that when performing an original version of the Stroop task [20], children tend not to experience interference until around age six due to a lack of automatic processing of written language. However, once children can automatically process written language, Stroop interference effects peak at 7–8 years of age and begin to decline from age 10 to 17 years, reaching adult-like levels of interference [21]. The attentional control ability necessary for performing tasks such as the Stroop task is also related to the development of the frontal area in the human brain [22,23,24]. Studies show that cognitive and attentional control in children (8–12 years) is less developed than in adults, with cognitive and behavioral inhibition improving in terms of accuracy and response times as children age. Effective attentional control in children is associated with neural activation of the left VLPFC, while in adults, it is associated with neural activation of the right VLPFC [22]. Activation of the DLPFC increases with age in children (aged 7–13 years) during the Stroop task [24]. As children mature, they can more effectively utilize the cognitive resources necessary to perform these tasks. These findings suggest that the frontal regions associated with attentional control continue to develop throughout childhood and adolescence.

According to the load theory of attention [8], a high perceptual load can reduce distractor interference during selective attention tasks in young adults. However, a recent study [25] demonstrated that children aged 7–8 years experienced a decrease in performance under perceptual load compared to a no-load condition, indicating a different pattern of load effects compared to young adults. Considering the developmental trajectories of WM and attentional control functions, it is plausible to hypothesize that school-aged children and adolescents would exhibit less mature interaction patterns between WM loads and attention compared to young adults, due to the ongoing development of these cognitive process.

Childhood and adolescents represent a period during which WM capacity and interference suppression develop both quantitatively and qualitatively alongside the maturation of related brain areas [4,18,22,26]. It is crucial for both children and adults to suppress undesired interferences and focus selectively on relevant tasks. Understanding the development of the interaction between WM and selective attention is particularly important for addressing and intervening in neurodevelopmental disorders such as ADHD, where difficulties in these cognitive functions are prevalent [27,28]. For instance, an increased WM load makes it more challenging to suppress task-irrelevant sounds in individuals with ADHD [29]. Additionally, as WM load and task complexity increase, hypoactivation was observed in both adults and adolescents (aged 12–23 years) in the right striatum, right cerebellum, and left occipital gyrus [30]. However, few studies have explored the interaction between WM and attention in typically developing children and adolescents [19,31]. Furthermore, to our knowledge, no research has examined the development of the interaction between WM and attention based on the overlapping cognitive resources involved in target or distractor processing, particularly by subdividing the WM and attention tasks.

Thus, in the current study, we investigated the interaction between WM and attention in typically developing school-aged children and adolescents (aged 10–14 years), whose WM subcomponents have begun to become independent [32] and for whom the Stroop effect has started to decrease to an adult-like level [21]. We also examined the interaction between these two systems in young adults and compared the patterns of interaction between the two age groups. Specifically, we employed task paradigms from Kim et al. [7] to examine the effects of WM load on selective attention.

In the previous study [7], the authors utilized WM tasks designed to engage either verbal or spatial short-term storage loads within the WM system. By incorporating these WM tasks into a dual-task paradigm, the previous study investigated whether a WM task requiring verbal or spatial short-term memory storage could either increase or decrease the Stroop interference effect. This effect depended on how the types of information in short-term memory storage shared resources with either target or distractor processing in the Stroop task. Using the same paradigm as Kim et al. [7], we expected to observe differentiated interaction patterns between WM and attention in young adults (aged 18–25 years) based on the resources shared between WM and attention processing. However, we hypothesized that school-aged children and adolescents may not benefit from WM loads that share resources with the distractor processing in the attention task, as their attentional control functions to inhibit unwanted information and subcomponents of WM are still developing [4,5,19]. In other words, as children age and their attentional control functions and executive control mechanisms mature, they may benefit from WM loads that require the same resources as interfering stimuli in the Stroop task.

## 2. Materials and Methods

### 2.1. Participants

Participants were divided into two primary age groups: school-aged children and adolescents (C/A; 10–14 years) and young adults (YA; 18–25 years). Sixty YA participants were recruited through offline and online advertisements around university campuses in Seoul, South Korea. Sixty-five school-aged C/A participants attending elementary or middle schools in Incheon, South Korea, were recruited through offline advertisements. All participants were provided with sufficient explanations about the study and provided written informed consent. In the case of C/A, both the legal guardian and child/adolescent provided consent for participation. YA participants received either an additional course credit or a gift certificate as a reward for their participation. C/A participants received a gift certificate and the result of the Multi-dimensional Learning Strategy Test-II (MLST-II) [33]. When obtaining consent, all YA participants reported no current or past neurodevelopmental/psychiatric conditions or diagnoses. Parent reports were used to screen for neurodevelopmental or psychiatric conditions in children and adolescents, and none reported any current or past psychiatric conditions. All the experimental protocols and methods were approved by the institutional review board at the university where this study was conducted (2017-003-002).

**Figure 1:**
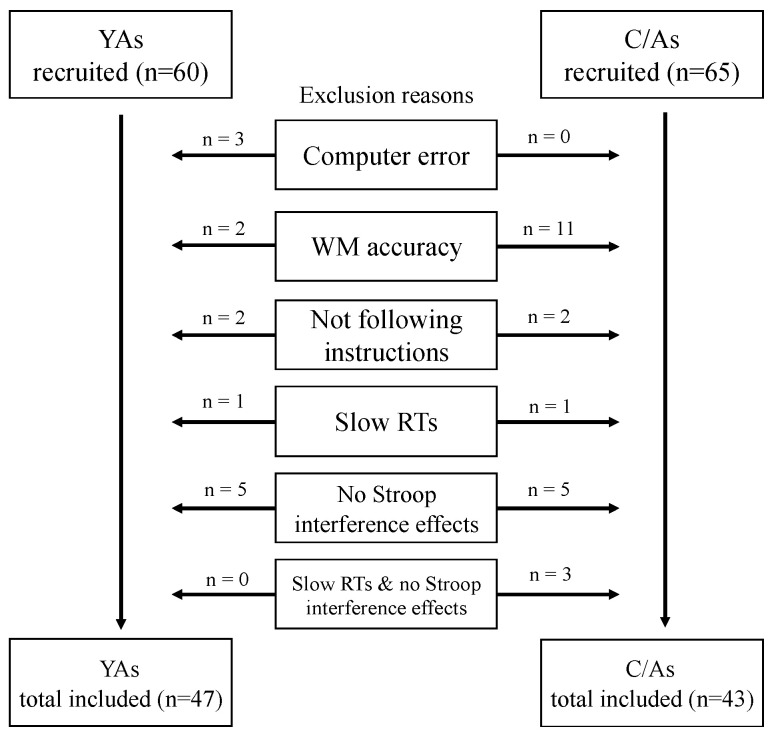
Flow of the inclusion and exclusion process for the final data.

Participants were excluded from the analysis if their accuracy and reaction times (RTs) did not meet the pre-set criteria (Figure 1). In YA participants, three participants stopped the experiment in the middle of the study due to a computer error. Additionally, two YAs were excluded as their accuracies in the WM task were below a chance level, and two others were excluded from the final analyses for not following the instructions. One YA was excluded due to slow RTs in the Stroop task (three standard deviations from the mean RTs), and five YAs were excluded for not exhibiting Stroop interference effects in the single-task condition. In children, 11 C/As were excluded due to low accuracy in the WM task, and one of those also showed low accuracy in the incongruent trials of the Stroop task, resulting in insufficient data for subsequent analyses. Additionally, two C/As were excluded for not following instructions, a C/A was excluded due to slow RTs in the Stroop task (three standard deviations from the mean RTs), and five C/As were excluded for not exhibiting Stroop interference effects in the single-task condition. Three C/As were excluded due to both low accuracy in the WM task and not exhibiting Stroop interference effects in the single-task condition. As a result, 47 YA and 43 C/A participants were included in the final analyses. The characteristics of the final participants included in the analyses in each WM task condition is presented in Table 1.

### 2.2. Apparatus and Stimuli

All tasks were presented on a 21.5-inch (1920 × 1080 resolution) computer (Apple Inc., Cupertino, CA, USA) running MATLAB (MathWorks Inc., Natick, MA, USA) and the Psychophysics toolbox. The distance between the participant and the monitor was about 60 cm.

The stimuli used in the left/right decision Stroop task were black arrows and words. The words “left” or “right” subtended a visual angle of 3.11° × 1.03°, and the arrows (“←” or “→”) had a visual angle of 4.14° × 1.03°. Arrows and words were presented 1.03° above or below the fixation point. The position of the arrow and word was presented randomly to prevent participants from staring at a particular area.

The stimuli used in the verbal WM (VWM) condition were Korean characters: “Ga”, “Na”, “Da”, “Ra”, “Ma”, “Ba”, “Sa”, “Ah”, “Ja”, “Cha”, “Ka”, “Ta”, “Pa”, or “Ha”. Seven out of these fourteen characters were chosen randomly and presented horizontally, each subtending a visual angle of 0.88° × 0.88°. In the recognition test trial, one character from the memory set was presented at the center of screen.

The stimuli used in the spatial WM (SWM) condition were black colored squares with a visual angle of 0.31° × 0.31°. The positions of four squares were presented randomly out of nine possible positions within a virtual circle centered on the fixation point. In the recognition test trial, a black empty square from the memory set was used.

### 2.3. Task Designs and Procedures

Participants were assigned into one of two WM dual-task experiments. All participants performed both Stroop-only and dual-task conditions. We utilized the same left/right Stroop task as Kim et al. [7]. In the previous study, the researchers demonstrated that the automatic processing of responding to the direction of an arrow presented above the fixation point interfered with the task at hand, which required responding to the meaning of the writing word (“Left” or “Right”). Specifically, the distracting information in this modified Stroop task necessitates spatial processing, whereas the target processing requires verbal processing. Additionally, we used the same WM tasks as in the previous study [7]: a verbal WM task requiring the maintenance of verbal information and a spatial WM task requiring the maintenance of spatial information.

**Figure 2:**
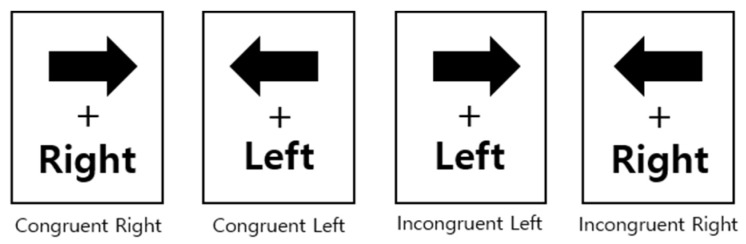
Stimuli in the left/right decision Stroop task.

In the Stroop only condition, participants performed the left/right decision Stroop task only. In the dual-task condition, participants performed the Stroop task while simultaneously completing a WM task (verbal or spatial). The Stroop task consisted of two congruency conditions. In the congruent condition, the direction of the arrow matched the meaning of the word (i.e., left or right), whereas in the incongruent condition, the direction of the arrow was presented in the opposite direction to the meaning of the word (Figure 2).

In the verbal WM dual-task experiment, participants were presented with an instruction to “Remember the characters” for 1500 ms at the beginning of each trial. This was followed by a black “+” fixation for 1000 ms, and then seven randomized characters were displayed at the center of the screen. After another 1000 ms of fixation, participants performed the left/right decision Stroop task. For the Stroop task, participants were instructed to ignore the direction of the arrow and judge the direction according to the meaning of the word. If the word was “Left”, they pressed the key with the “←” sticker using the index finger of their right hand. If the word was “Right”, they pressed the key with the “→” sticker using the middle finger of their right hand. The Stroop stimuli remained on the screen until the participant responded, followed by a 500 ms fixation screen. After completing the Stroop task, one character from the memory set was presented at the center of the screen as a WM recognition trial. Participants judged whether it was one of the seven previously memorized characters. If it was a memorized character, they pressed a keyboard button with an “O” sticker using the index finger of their left hand. If it was not, they pressed a keyboard button with an “X” sticker using the middle finger of their left hand. The character remained on the screen until the participant responded, followed by the next trial. The to-be-memorized set of words and the memory-probe in the WM task were updated on a trial-by-trial basis to ensure that participants engaged in active maintenance within their WM system. To prevent any reliance on familiarity processing, there was no repetition of the same list in consecutive trials.

**Figure 3:**
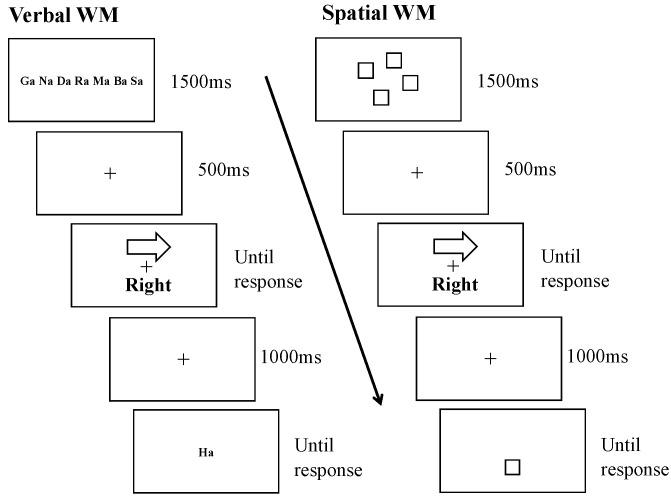
Examples of dual-task conditions in each WM condition (left: VWM; right: SWM).

The spatial WM dual-task experiment was identical to the verbal WM one, except that black squares were used instead of characters. In the WM array, four black squares appeared on the screen, and participants were instructed to remember their spatial positions while performing the Stroop task. In the WM recognition trial, a black blank square was presented, and participants judged whether its position was one of the four previously memorized positions. Like the VWM task, the to-be-memorized locations and the memory-probe in the SWM task were updated on a trial-by-trial basis. There was no repetition of the same locations in consecutive trials in the SWM task. Examples of the experimental procedures of the two dual-task experiments are illustrated in Figure 3.

The Stroop-only condition was identical to the dual-task condition, except a blank screen was presented for 500 ms instead of the WM array. The dual-task condition comprised four practice trials and 20 experimental trials, with a 50% probability of congruent and incongruent conditions. The WM test also had a 50% probability of retrieval from the memory set. In the Stroop-only condition, participants performed 20 left/right decision Stroop trials. The order of the Stroop-only condition and the dual-task condition was counterbalanced across participants.

### 2.4. Data Analysis

Participants’ mean accuracy and median response times (RTs) were analyzed using SPSS 20.0. Only the RTs of correctly responded trials were included for both Stroop and WM tasks. Data that did not meet the pre-set criteria based on the previous study [7] were excluded: RTs that were under or over three standard deviations from the average, an accuracy below 50% (both the Stroop and WM tasks), and participants who did not show Stroop interference effects by reacting faster to the incongruent condition than the congruent condition in the Stroop-only condition.

Statistical analyses were conducted to examine participants’ age, gender, accuracies in the WM and Stroop tasks, and RTs in the Stroop task. To address our research questions, a mixed-design repeated measures ANOVA was conducted on the Stroop task RT data in each experiment: 2 (group: YAs, C/As; between-subjects) × 2 (task: single, dual; within-subjects) × 2 (congruency: congruent, incongruent; within-subjects). When significant effects were observed, a 2 (task: single, dual; within-subjects) × 2 (congruency: congruent, incongruent; within-subjects) repeated measures ANOVA was performed on the Stroop RT data in each age group to test whether the overlapping effects of WM load and target/distractor processing in the attention task varied across the age groups.

## 3. Results

### 3.1. Experiment 1: VWM Dual-Task

A 2 (group) × 2 (task) × 2 (congruency) repeated measures ANOVA on accuracy data in the Stroop task revealed a significant main effect of congruency (*F*(1, 46) = 17.73, *p* < 0.001, *η*^2^ = 0.28). Specifically, all participants showed a lower accuracy in the incongruent condition (97.1%) than in the congruent condition (99.8%), regardless of the single- or dual-task load. No other interactions or main effects were significant (all *p*s > 0.05) (Table 2).

The same ANOVA conduced on the RT data in the Stroop task revealed significant main effects of task, congruency, and age (task: *F*(1, 46) = 45.48, *p* < 0.001, *η*^2^ = 0.50; congruency: *F*(1, 46) = 59.63, *p* < 0.001, *η*^2^ = 0.57; age: *F*(1, 46) = 10.62, *p* < 0.005, *η*^2^ = 0.19) (Table 3). None of the interaction effects were significant.

**Figure 4:**
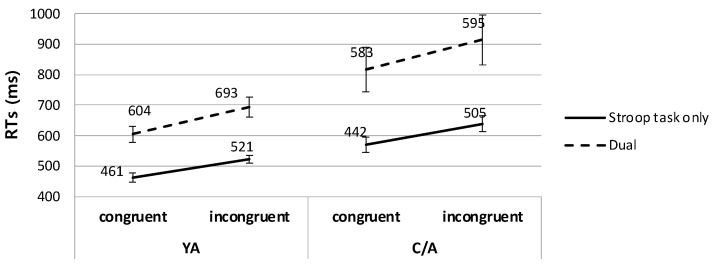
Stroop interference effects of each age group in the VWM dual-task experiment.

To test the significant effect of WM task load in each age group, we conducted a 2 (task) × 2 (congruency) repeated measures ANOVA on the RT data in the Stroop task for each group. In the YA group, the main effects of task and congruency were significant (task: *F*(1, 24) = 53.18, *p* < 0.001, *η*^2^ = 0.69; congruency: *F*(1, 24) = 57.56, *p* < 0.001, *η*^2^ = 0.71). Importantly, the interaction between the two factors was also significant (*F*(1, 24) = 5.15, *p* < 0.05, *η*^2^ = 0.18), indicating greater Stroop interference effects in the dual-task condition with the VWM task (89.6 ms) than in the Stroop-only condition (60.0 ms). That is, our results replicated previous findings in young adults [7], showing increased Stroop interference effects when the resources used in the WM load and the resources for target processing in the attention task overlapped. For the C/A group, the same two-way repeated measures ANOVA revealed the main effects of task (*F*(1, 22) = 18.77, *p* < 0.001, *η*^2^ = 0.46) and congruency *F*(1, 22) = 20.26, *p* < 0.001, *η*^2^ = 0.48). However, an interaction between the two factors was not significant in child and adolescent participants (*F*(1, 22) = 0.68, *p* = 0.42, *η*^2^ = 0.03), indicating that Stroop interference effects were not significantly changed under the WM load compared to the load-absence condition, despite the overlapping resources required in both the WM task and the target processing in the attention task. Figure 4 depicts the RT results in the Stroop task for each group in Experiment 1.

### 3.2. Experiment 2: SWM Dual-Task

A 2 (group) × 2 (task) × 2 (congruency) repeated measures ANOVA on accuracy data in the Stroop task revealed significant main effects of task (*F*(1, 40) = 14.19, *p* < 0.001, *η*^2^ = 0.26) and congruency (*F*(1, 40) = 8.76, *p* < 0.01, *η*^2^ = 0.18). Specifically, all participants showed a lower accuracy in the incongruent condition (97.6%) than in the congruent condition (99.5%), regardless of the single- or dual-task load. Additionally, participants performed significantly better under the SWM load (99.4%) than in the load-absence condition, regardless of congruency. Lastly, significant interaction between task and congruency was also found (*F*(1, 40) = 8.67, *p* < 0.01, *η*^2^ = 0.18). No other interactions or main effects were significant (all *p*s > 0.05) (Table 4).

Next, ANOVA results on the RT data showed significant main effects of task (*F*(1, 40) = 112.03, *p* < 0.001, *η*^2^ = 0.74), congruency (*F*(1, 40) = 36.75, *p* < 0.001, *η*^2^ = 0.48), and age (*F*(1, 40) = 15.54, *p* < 0.001, *η*^2^ = 0.28) (Table 5). Additionally, two-way interaction effects of task and age (*F*(1, 40) = 5.29, *p* < 0.05, *η*^2^ = 0.12) and congruency and age (*F*(1, 40) = 5.20, *p* < 0.05, *η*^2^ = 0.12) were significant. Notably, a three-way interaction of task, congruency, and age was significant (*F*(1, 40) = 5.22, *p* < 0.05, *η*^2^ = 0.12), indicating differentiated effects of WM load on selective attention depending on the age group.

**Figure 5:**
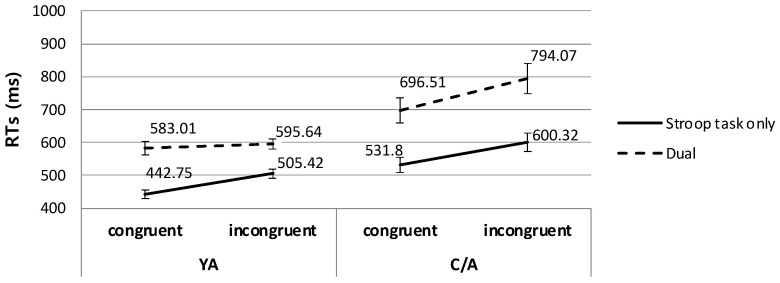
Stroop interference effects of each age group in the SWM dual-task experiment.

To further investigate the three-way interaction, 2 (task) × 2 (congruency) repeated measures ANOVAs on the RT data in the Stroop task were conducted for the YA and C/A groups. First, results for the YA group replicated previous findings [7]. Specifically, we found significant main effects of task (*F*(1, 21) = 70.78, *p* < 0.001, *η*^2^ = 0.77) and congruency (*F*(1, 21) = 34.29, *p* < 0.001, *η*^2^ = 0.62). Importantly, an interaction effect was also significant (*F*(1, 21) = 7.43, *p* < 0.05, *η*^2^ = 0.26; Figure 5). Post hoc *t*-tests on the interaction effect revealed that YA participants showed a typical Stroop interference effect in the Stroop-only condition (*t*(21) = 7.63, *p* < 0.001). However, the interference effect disappeared when the WM task required the same spatial resources as those required in distractor processing in the attention task (*t*(21) = 0.93, *p* = 0.36).

This pattern, however, was absent in the child and adolescent group. Specifically, the same ANOVA revealed significant main effects of task (*F*(1, 19) = 51.36, *p* < 0.001, *η*^2^ = 0.73) and congruency (*F*(1, 19) = 17.82, *p* < 0.001, *η*^2^ = 0.48). However, an interaction effect between the two factors was not significant in the C/A group (*F*(1, 19) = 0.92, *p* = 0.35, *η*^2^ = 0.05). That is, unlike young adults, children and adolescents showed significant interference effects even when the concurrent WM task required the same resources as those for the distractor processing in the selective attention task (in the Stroop-only condition: *t*(19) = 6.72, *p* < 0.001; in the dual-task condition: *t*(19) = 2.91, *p* < 0.005).

### 3.3. WM Performance

To compare the task difficulty in the VWM and SWM tasks, we conducted independent samples *t*-tests on performance in each age group in the two experiments. For the YA group, the accuracy of the VWM task (88.4%) was significantly higher than that of the SWM task (83.6%) (*t*(42.29) = 2.10, *p* < 0.05). However, the RT data showed the opposite pattern. Specifically, the RTs were significantly faster in the SWM task (941 ms) compared to the VWM task (1197 ms) in the YA group (*t*(43.38) = 3.79, *p* < 0.001). Taken together, the performance on the two WM tasks demonstrated a speed–accuracy trade-off, making it difficult to determine differences in task difficulty between the two WM tasks. Similarly, we did not find any performance differences in the C/A group between the two WM tasks. Specifically, differences between accuracies of the VWM task (79.8%) and the SWM task (84%) in the C/A group were not significant (*t*(41) = 1.46, *p* = 0.15). Lastly, the independent samples *t*-test on the RT data of the two WM tasks showed insignificant differences in the C/A group (for VWM: 1337 ms; for SWM: 1245 ms; *t*(41) =.84, *p* = 0.40).

In addition, we conducted independent samples *t*-tests on WM accuracies and RT data using the “age” as a factor to compare performance differences in WM between the YA and C/A groups. First, for the VWM, we found a significant difference in WM accuracy between the two age groups (*t*(46) = 3.03, *p* < 0.05), indicating adults performed better in the VWM task than children and adolescents. The same tests on the VWM RT data showed insignificant results (*t*(46) = −1.52, *p* = 0.13). Next, for the SWM, the difference in WM accuracy was not significant between the two groups (*t*(40) = −0.34, *p* = 0.74). However, adults responded significantly faster than children and adolescents in the SWM task (*t*(28.77) = −3.41, *p* < 0.05).

### 3.4. Correlations between Age and the Stroop Interference Effects under WM Loads

**Figure 6:**
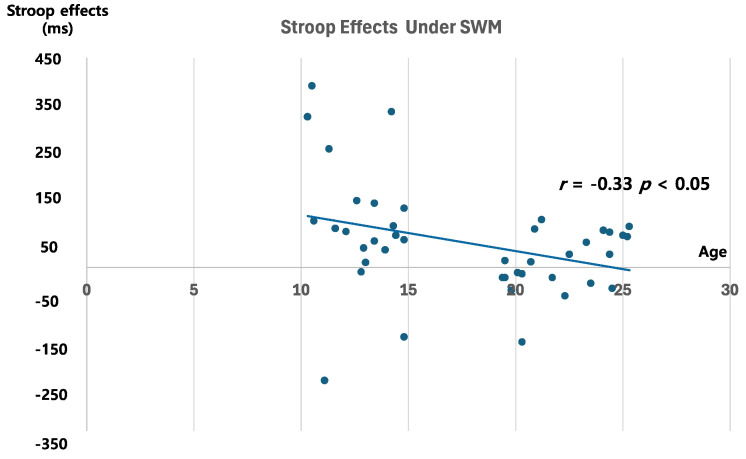
Correlation between age and Stroop interference effects in the SWM dual task.

To test developmental changes in the interaction between attention and WM based on shared resources between the two systems, we conducted correlational analyses using the Stroop interference scores in each task condition in relation to participants’ age. The Stroop interference scores were computed by subtracting the RTs in the congruent condition from the RTs in the incongruent condition. In Experiment 1, where the VWM load was imposed, age showed no significant correlation with the interference scores in either the single- or dual-task conditions (*r* = −0.18, *p* = 0.21 and *r* = −0.08, *p* = 0.58, respectively). However, in Experiment 2, where the SWM load was imposed, age and the interference score under the dual-task condition were significantly correlated (*r* = −0.33, *p* < 0.05; Figure 6). The significant negative correlation suggests that the interference effect under the WM load, where the WM task shares resources with distractor processing in the attention task, decreases as participants’ ages increase. However, this relationship was not found in the single-task condition (*r* = −0.17, *p* = 0.28), indicating developmental changes in the interaction between attention and WM functions as individuals progress from school-age childhood to young adulthood.

## 4. Discussion

The purpose of this study was to investigate the effects of WM load on Stroop interference effects in young adults (YAs) and children/adolescents (C/As), based on overlapping resources between WM and attention tasks. Replicating previous findings [7], YAs exhibited increased Stroop effects when the WM load and the resources for target processing overlapped. Conversely, they showed significantly reduced interference effects when the WM task and distractor processing in the attention task shared cognitive resources. These differentiated effects of WM loads on attention were not observed in children and adolescents aged 10–14 years. Specifically, interaction effects were absent in the C/A group, regardless of overlapping resources between the two cognitive systems.

Our findings on young adults are consistent with previous research suggesting that selective attention may not be disturbed unless it shares resources with the concurrent WM load [7,9,13,14,34]. However, some studies have failed to replicate Kim et al.’s [7] findings, showing that attentional processing remains unaffected by WM load even when the load utilizes the same resources as distractor processing in the attention task [34]. It is noteworthy that the accuracy levels of the WM task in Gil-Gómez de Liaño et al. [34] are relatively low compared to other studies using the same memory task [7]. Specifically, the accuracy level of the WM task in Gil-Gómez de Liaño et al.’s study [35] was only 61%, whereas accuracies for the same spatial WM task were 85% in Kim et al.’s study [7] and 83.2% in young adult participants of the current study. As discussed in Gil-Gómez de Liaño et al.’s study [35], it is plausible that the spatial WM task was too difficult for their participants, leaving too few resources for distractor processing in the attention task, resulting in no effects of WM load on the attention task. Future research, including various difficulty levels of WM loads, can elucidate the appropriate levels of WM loads needed to reduce distracting effects in the attention task when the WM load shares resources with distractor processing.

The C/A participants in the present study exhibited a lack of interaction between attention and WM, regardless of the cognitive resources shared between the two systems. This finding contrasts with those observed in YAs. However, a previous study on adolescents (12–16 years) demonstrated similar results to those in our C/A group regarding the interaction effects between attention and WM [31]. Specifically, Spronk and Jonkman [31] investigated the effects of concurrent WM loads on attentional control in adolescents and adults using a word–face Stroop task, where face stimuli served as distractors. Employing both behavioral and electrophysiological measures, they found that only adult participants showed an increased event-related potential (ERP) component for conflict resolution when WM loads were imposed during the Stroop task. In contrast, adolescents exhibited an already heightened ERP component even without the WM load, and these components remained unchanged with the concurrent WM load. Spronk and Jonkman concluded that the attentional control mechanism in adolescents was immature and still developing, leading to a lack of interaction effect by WM loads [31].

Our findings are also consistent with broader developmental patterns of WM. Studies indicate a non-linear growth pattern in WM, with rapid development during childhood, a brief acceleration in early adolescence, and stabilization in late adolescence [36]. This suggests that WM capacity undergoes significant changes during these developmental periods, aligning with the observation that attentional control mechanisms might not fully integrate with WM in younger populations. Furthermore, the current findings for children and adolescents aged 10 to 14 years provide new evidence on the development of the interaction between attention and WM based on the cognitive resources available to individuals. Notably, children exhibited significantly slower reaction times (RTs) compared to adults in the Stroop task (congruent condition: t(66.84) = 5.19, *p* < 0.001; incongruent condition: t(63.48) = 4.99, *p* < 0.001), supporting the notion of immature attentional control functions in younger participants. This inefficiency in attentional control may contribute to the lack of interaction effects between attention and WM in the C/A group. These developmental findings suggest that the cognitive resources necessary for the interaction between attention and WM are still developing in children and adolescents, potentially accounting for the observed differences compared to order individuals.

It is noteworthy that the difficulty level of the WM tasks, especially verbal, was not comparable between the two age groups in the present study. The independent *t*-tests on the accuracy data in the VWM task revealed significantly better performance levels in the YA group compared to the C/A group. This raises the question that more cognitive load was imposed on children and adolescents than on young adults during the dual task, potentially resulting in different effects of the WM load between the two groups. However, if this were the case, the C/A group should have shown much greater interference effects than the YA group, as the VWM task would have required more cognitive resources from participants in the C/A group. Contrary to this expectation, we found that only the YA group, not the C/A group, exhibited more interference effects when the concurrent WM load (i.e., verbal) shared resources with the target processing (i.e., verbal) in the Stroop task. Our results in Experiment 2 with the SWM task support this idea, suggesting that differences in WM task difficulty may not be responsible for the different effects of WM loads between the two age groups. Specifically, even when the difficulty level of the WM task was comparable between the two age groups, a significant interaction between the age, task, and congruency factors was found. This interaction indicated that only the YA group showed significantly reduced interference effects in the Stroop task when the WM task required the same type of resources as the distractor processing in the attention task. Hence, although it is important to consider the level of task difficulty between the two age groups, in our study, the task difficulty itself cannot account for the different interaction patterns between WM and attention between the YA and the C/A groups. Future research with equivalent levels of difficulty in WM tasks for children and adults may more clearly elucidate the effects of WM loads on attention in developing individuals.

In fact, both WM and attentional control functions are involved in executive functions. Components of executive functions follow different developmental trajectories [37], and until the age of 11, these components act as elements of a single executive function rather than independently [38]. Miyake and colleagues conducted a confirmatory factor analysis using various tasks to measure executive functions and concluded that executive functions consist of three elements: inhibition, shifting, and WM [39,40]. These components are distinguishable from one another yet interact closely. This framework has been supported by studies on children and adolescents [38,41]. Additionally, Karr and colleagues’ systematic review and re-analysis of latent variable studies on the diversity of executive functions reported that executive functions primarily consist of three components: inhibition, WM, and shifting. Their findings confirmed that from school-age childhood (6–12 years) to adolescence (13–17 years), executive functions develop distinctly, with shifting emerging as a critical component during this developmental period [42]. However, it remains unclear whether these three components function together or independently in children as they do in adults. For example, Lehto et al. found that for children aged 8 to 13 years, a model with high correlations among inhibition, WM, and shifting was statistically significant, similar to adults [41]. Conversely, Brydges et al. demonstrated that for children aged 9–11 years, executive function was better explained as a single entity rather than three distinct components [38]. Although results may vary based on the specific tasks and age ranges used to measure each factor, it is generally agreed among researchers that these components continue to develop throughout childhood [42,43,44,45]. Thus, unlike adults who can effectively allocate resources for goal-directed behavior using fully developed executive functions, children may struggle to manage these resources effectively due to ongoing development. Consequently, selective attention may not be significantly affected by WM load in children and adolescents in the same manner as in young adults.

This study has some limitations. First, the memory task was not measured separately. That is, the accuracy of WM was measured only in the dual-task conditions. If we had measured WM abilities in our participants independently, we could have explored the effects of concurrent attentional load on memory performance based on shared resources in both YA and C/A groups. Although we cannot measure the effect of attention on WM in our paradigm, we expect differential effects of attention on WM tasks based on how resources are shared between the two systems only in adult participants. Second, the high accuracy levels in the Stroop task could indicate a ceiling effect, which may have obscured the effect of WM load on accuracies. However, a previous study by Kim et al. [7], which observed a reduction in the Stroop effect due to WM load using the same task as in our study, also showed the similar levels of accuracy (Stroop-only condition: 97.27%, 100%; dual condition: 99.55%, 98.5%; congruent condition: 99.32%, 100%; incongruent condition: 97.5%, 98.5%, respectively in the current study and the one by Kim et al. [7]). In a future study, it would be beneficial to control the difficulty levels of the task to confirm the effect of WM load on selective attention in both RTs and accuracies. Third, the age range of our C/A participants was 10–14 years, encompassing both children and adolescents. Although some studies on cognitive development refer to participants aged 4–15 as children [46], it is important to note that this group may not be completely homogeneous due to the inclusion of both children and adolescents. The increased variability in the developmental data may have contributed to the absence of significant interactions observed in the C/A group, likely due to the inherent differences in cognitive development among children and adolescents. Given the small number of participants in the 10–11-year age range (“children”, N = 6), it was not feasible to conduct a three-group analysis (i.e., young children, adolescents, young adults) in the current study. Instead, we conducted correlation analyses to directly examine the relationship between age and changes in Stroop interference effects modulated by the WM load. Future research with a sufficient sample size and a greater number of trials in the Stroop task could reduce the variability of the results, providing valuable insights into the developmental differences in the interaction between attention and WM among young children, adolescents, and young adults. Lastly, while our study aimed to recruit healthy participants through community advertisements, it is important to acknowledge that community recruitment alone does not guarantee the absence of neurodevelopmental or psychiatric conditions. Although we conducted preliminary screenings to ensure participants reported no current or past neurodevelopmental or psychiatric conditions, and parents confirmed the same for child and adolescent participants, we did not administer comprehensive clinical assessments to verify these self-reports. While previous studies with neurotypical young adult participants on their interaction patterns between WM and attention also recruited community samples without direct assessments, this limitation should be considered when interpreting the results. Future studies could benefit from incorporating detailed clinical evaluations to more rigorously exclude participants with underlying conditions.

Despite these limitations, the current study provides important insights into the developmental trajectories of the effects of WM load on attentional functions. Previous studies have explored the relationship between perceptual loads and selective attention in children [47], but research on the relationship between WM load and selective attention in children and adolescents is limited. The current study provides important insights into the developmental trajectory of the interaction between WM load and selective attention. While our findings reveal differing patterns between adults and children/adolescents, they highlight how the nature of this interaction evolves with age. Our results emphasize the developmental differences observed, suggesting that the interaction between WM load and selective attention changes significantly from childhood to adulthood. In addition to insights into neurotypical development, our findings provide foundational knowledge for understanding various disorders that exhibit specificity in attention mechanisms. For example, attention deficit hyperactivity disorder (ADHD) is characterized by impairments in attention mechanisms, such as response inhibition, attentional control, and selective attention. Individuals with ADHD may be deficient in tasks requiring selective attention and response inhibition, such as the Stroop or flanker tasks, due to their inability to allocate limited attentional resources effectively [27]. Deficits in selective attention observed in the Stroop task are consistently seen in children with ADHD, with interference effects being stronger in this group compared to typically developing children [48,49].

Our study found that while young adults exhibited reduced Stroop interference effects under the WM load requiring the same cognitive resources as distracting information in the Stroop task, children and adolescents did not show similar effects. This suggests that the interaction between WM load and selective attention evolves with age. The positive effects observed in young adults indicate potential intervention strategies for improving selective attention in individuals with ADHD. However, given that children and adolescents did not demonstrate the same effects, it is crucial for future research to explore age-appropriate interventions that account for developmental differences. Thus, interventions for children/adolescents with ADHD should be designed considering these developmental trajectories. Our findings provide a basis for developing strategies to improve selective attention abilities in individuals with ADHD.

Interestingly, we found that typically developing children/adolescents under the age of 14 did not show the same interaction between WM and selective attention as young adults. However, it is possible that gifted children, whose WM capacity is larger and in whom resource distribution is more efficient [50], may show different results from those observed in typically developing ones. For example, because gifted children have larger WM resources, they might retain cognitive capacity even after a WM load, allowing resources for distractor processing, and thus the Stroop effect might be reduced when the resources for WM load and distractor processing overlap. That is, unlike typically developing children, gifted children whose WM capacities are relatively larger, would exhibit similar interaction patterns of attention and WM to those in adults. Overall, it is necessary to investigate the mechanism of attention according to WM load in groups that exhibit specific attention and/or memory functions, such as those with ADHD or gifted children.

## 5. Conclusions

The current study aimed to explore the effect of WM load on selective attention in typically developing children and adolescents, as well as neurotypical young adults. In young adults, the Stroop effect increased under the dual-task condition compared to the Stroop-only condition when the WM resources and the resources for target processing overlapped. Conversely, the Stroop effect decreased when the resources for distractor processing overlapped with WM resources. However, no interaction between WM and selective attention was observed in children and adolescents. In other words, even when WM resources overlapped with those required for target or distractor processing, the magnitude of the Stroop interference effect remained unchanged in the C/A group. These results provide fundamental developmental data on how limited cognitive resources are utilized and how WM and attention interact during development. Understanding these interactions in typically developing populations not only deepens our knowledge of cognitive development but also has significant practical implications. For instance, this research can inform the design of educational strategies that consider the limitations of WM and attention in children and adolescents. Additionally, it offers insights that can guide the development of tailored interventions for children with atypical cognitive development, such as those with neurodevelopmental disorders or those who are gifted. By understanding how their WM and attention processes differ from typical developmental trajectories, more effective educational tools, therapeutic interventions, and cognitive training programs can be designed to meet the specific needs of diverse learners.

## Figures and Tables

**Table 1:** Task assignment and participant demographics.

WM Task	Age Group	Sex		Age
	N	N		M(SD)
Verbal (N = 48; Experiment 1)	YAs (N = 25)	Male: 16	Female: 9	21.3 (2.2)
	C/As (N = 23)	Male: 9	Female: 14	12.8 (1.4)
Spatial (N = 42; Experiment 2)	YAs (N = 22)	Male: 15	Female: 7	22.2 (2.1)
	C/As (N = 20)	Male: 10	Female: 10	12.8 (1.5)

**Table 2:** Three-way repeated ANOVA (accuracy) in Experiment 1 (VWM load).

	*F*	*p*	*η* ^2^
Task	*F*(1, 46) = 0.358	0.55	0.01
Congruency	*F*(1, 46) = 17.73	<0.001	0.28
Age	*F*(1, 46) = 2.00	0.16	0.04
Task **X** Age	*F*(1, 46) = 0.05	0.83	0.00
Congruency X Age	*F*(1, 46) = 1.79	0.19	0.04
Task X Congruency	*F*(1, 46) = 0.03	0.86	0.00
Task X Congruency X Age	*F*(1, 46) = 0.22	0.64	0.01

**Table 3:** Three-way repeated ANOVA (RTs) in Experiment 1 (VWM load).

	*F*	*p*	*η* ^2^
Task	*F*(1, 46) = 45.48	<0.001	0.50
Congruency	*F*(1, 46) = 59.63	<0.001	0.57
Age	*F*(1, 46) = 10.62	<0.005	0.19
Task **X** Age	*F*(1, 46) = 2.80	0.10	0.06
Congruency X Age	*F*(1, 46) = 0.14	0.71	0.00
Task X Congruency	*F*(1, 46) = 2.56	0.12	0.05
Task X Congruency X Age	*F*(1, 46) < 0.001	0.99	0.00

**Table 4:** Three-way repeated ANOVA (accuracy) in Experiment 2 (SWM load).

	*F*	*p*	*η* ^2^
Task	*F*(1, 40) = 14.19	<0.001	0.26
Congruency	*F*(1, 40) = 8.76	<0.01	0.18
Age	*F*(1, 40) = 0.28	0.60	0.01
Task **X** Age	*F*(1, 40) = 2.15	0.15	0.05
Congruency X Age	*F*(1, 40) = 0.02	0.89	0.00
Task X Congruency	*F*(1, 40) = 8.67	<0.01	0.18
Task X Congruency X Age	*F*(1, 40) = 0.08	0.78	0.00

**Table 5:** Three-way repeated ANOVA (RTs) in Experiment 2 (SWM load).

	*F*	*p*	*η* ^2^
Task	*F*(1, 40) = 112.03	<0.001	0.74
Congruency	*F*(1, 40) = 36.75	<0.001	0.48
Age	*F*(1, 40) = 15.54	<0.001	0.28
Task **X** Age	*F*(1, 40) = 5.29	<0.05	0.12
Congruency X Age	*F*(1, 40) = 5.20	<0.05	0.12
Task X Congruency	*F*(1, 40) = 0.37	0.55	0.01
Task X Congruency X Age	*F*(1, 40) = 5.22	<0.05	0.12

## Data Availability

Individual data are protected and thus are not shareable.
